# The hard and complex work of implementing new multi-agency risk assessment approaches to policing domestic abuse

**DOI:** 10.1057/s41300-023-00175-3

**Published:** 2023-04-05

**Authors:** Pamela Davies, Charlotte Barlow, Rebecca Fish

**Affiliations:** 1 Department of Social Sciences, Northumbria University, London, UK; 2 School of Justice, University of Central Lancashire, Preston, UK

**Keywords:** Domestic abuse, Multi-agency, Partnership working, Risk assessment, MARAC, Policing

## Abstract

In the period since multi-agency working became the dominant approach to tackling domestic abuse, there has been ongoing development and innovation. However, little is known about what tends to enhance or inhibit the roll-out of such initiatives. This article examines the process of building an enhanced flagship multi-agency model for policing domestic abuse. We report on results from semi-structured interviews, observations of meetings and an online survey with stakeholders who were involved in the development of the principles underlying a new multi-agency risk assessment conference (MARAC) process. The participants representatives from policing, third sector, health, and probation organisation-described positive benefits of the process, whilst challenges coalesced around the focus on and engagement of perpetrators, and the problem of assessing the dynamics of risk.

## Introduction

As we move through the fourth decade of policing domestic abuse (DA) through multi-agency working in the UK, it is clear that this approach to partnership working remains at the heart of state supported strategies for tackling such abuse. The introduction of the 2004 Domestic Violence, Crime and Victims Act established Multi-Agency Risk Assessment Conferences (MARACs) as a way to form a coordinated response to address high-risk cases of DA. UK DA charity Safe Lives define a MARAC as:

[…] a meeting where information is shared on the highest risk domestic abuse cases between representatives of local police, health, child protection, housing practitioners, Independent Domestic Violence Advisors (IDVAs), probation and other specialists from the statutory and voluntary sectors. (SafeLives 2014)

The key purposes of a MARAC are: information sharing, coordinated safety, and action planning linking with other relevant agencies (McLaughlin et al. 2018). SafeLives goes on to say:

At the heart of a MARAC is the working assumption that no single agency or individual can see the complete picture of the life of a victim, but all may have insights that are crucial to their safety. The victim does not attend the meeting but is represented by an IDVA who speaks on their behalf (SafeLives 2014).

Steele et al. (2011) review of MARACs show that core factors that are key to their effectiveness are: enhanced information sharing; appropriate agency representation; and the role of the IDVA in representing and engaging the victim in the process. Factors which were seen as supporting effective practice included having strong partnership links (including a commitment from agencies to tackle DA in general), strong leadership, good co-ordination through designated roles, and the availability of training and induction.

Although there are around 300 MARACs in England and Wales today, many are struggling to deal with the increasing number of cases referred to them. Efforts to improve the efficient targeting of the most prolific offenders have seen several multi-agency innovations to reduce risks to DA (Shorrock et al. 2020). Evaluations of such innovations have proliferated as the number and variety of approaches has increased. However, there has been little published on the amassed and collective findings. Indeed, it is hard to compare and contrast the common challenges and barriers to effective working, and glean robust evidence of what works in the search for best practice, in light of the variations in the approach taken. For example, one review of 22 published evaluations examined DA interventions across the UK to identify emerging good practice in multi-agency early intervention (Cleaver et al. 2019). The findings reveal a range of strategies and interventions that have been piloted and tested with varying degrees of success, but the authors found much variation in what constitutes ‘early intervention;’ therefore, the studies qualifying for inclusion in the review were only loosely similar. A further example of the difficulties in selecting a sample of case studies of multi-agency partnership working in the context of the policing of DA featured in the research by Davies and colleagues (Davies 2021; Davies et al. 2020). They had difficulty in selecting cases which measured up to even the most relaxed of qualification criteria.

One way of generating more robust and meaningful comparisons of success in achieving innovative practice is through gathering data on experiences of participation in evolving multi-agency approaches. Process evaluations are sometimes able to elicit such in-depth narratives. Experiential evidence has increasingly been recognised as an important set of knowledge of the craft of policing (Fleming 2015, Fleming and Rhodes 2018). We suggest that exploring the professional experiences of partners in multi-agency policing deepens our understanding of what helps and hinders the implementation of new processes and innovative practice. It is important to add experiential understanding into the evidence base about multi-agency working and innovation. Thus, with a view to improving planning for implementation and practice in the future, we foreground experiential evidence from a key stakeholder group involved in one such endeavour.

In this article we argue that to date, little is known about what tends to promote and inhibit the roll-out of innovations to the MARAC approach that nevertheless have a common shared vision amongst all stakeholders. We consider the difficulties in building a flagship multi-agency model for policing DA. Drawing on findings from research conducted of one recent streamlined approach and the wider literature base, we unpick the implementation problem and the complex work of introducing new approaches to multi-agency policing of DA. The issues discussed in this paper have international implications, given the increased usage of this model across the globe.

## Multi-agency risk assessment conferences: an overview

Prior to the 1990’s, multi-agency working within the context of DA was relatively neglected (Sampson et al. 1988). In the UK, the Home Office Circular 60 (1990) was influential in re-orientating multi-agency partnerships towards DA with the 1998 Crime and Disorder Act adding further stimulus to the development of multi-agency working. However, it was not until the 2004 Domestic Violence, Crime and Victims Act that this was implemented on a larger scale (Cleaver et al. 2019). This legislation, among other key changes, introduced MARACs. In England and Wales, the MARAC model developed primarily from an initiative introduced in 2003 in Cardiff. This initiative brought together a wide range of agencies including police, probation, local authorities, health, housing, refuge and what was then the Women’s Safety Unit. A process and outcome evaluation of its work pointed to the positive effects that this kind of multi-agency working had for victim-survivors of DA, particularly high-risk victims, and led to this multi-agency model being adopted throughout England and Wales (Robinson and Tregidga 2007). However, questions were raised regarding information sharing within the context of MARACs and the extent to which such conferences facilitated victim-survivor empowerment (Westmarland 2011; Walklate et al. 2021).

Cleaver et al. (2019) describe further challenges in terms of funding and resources (including availability of staff), competing organisational priorities (including the issue of working in professional “silos”) and the challenge of hierarchical relationships in which the police frequently feature as the lead agency. Issues have also been raised regarding how cases are selected to go to MARACS (i.e. which cases are included and excluded), limited understandings of the fluctuating nature of risk in DA cases (Barlow and Walklate 2021) and high volumes of cases making work load and safety planning difficult for staff to manage (Steele et al. 2011).

Walklate et al. (2021) provide a useful summary, outlining four issues that present ongoing challenges for MARACs. These are the appropriate identification of (high-risk) cases; the representation of appropriate agencies at these conferences; managing the volume of work; and appropriate action planning. Robbins et al. (2014) had previously listed amongst the challenges the varying ways in which agencies construct the victim, the practice implications of this, and the capacity of MARACs to recognise the complex lives of service-users. This is a finding later echoed by Shorrock et al. (2020) in their work on the role of multi-agency safeguarding hubs (MASH) and responding to the problem of repeat victimisation.

Awareness of these criticisms has led to regional and local areas in England and Wales re-designing their MARAC arrangements in recent years. During the Covid-19 pandemic, this included moving MARAC meetings to take place online (Walklate et al. 2021). For others, these criticisms resonate with professional stakeholder experiences of being engaged in MARAC processes. Many professionals have several years’ experience in being involved in decision-making on multi-agency panels and their contributions have led to incremental developments to work more effectively and holistically with individuals and families. This paper focuses on one such example of this approach to innovative practice.

## Innovating the MARAC process

This study explores a streamlined way of managing and coordinating the MARAC, which, in this article, we call ‘the new process’, implemented in one region in England. This process was designed to be sensitive to the underlying and complex interplay of factors and dynamics that give rise to DA. The aim is to provide holistic support for victims, children and perpetrators. In this article, we refer to this as ‘the whole family’. The new process does not rely on a single meeting like the traditional MARAC process, but rather includes four steps: gathering and assessing information, analysing risks and needs, identifying solutions and finally completing the case. The process prioritises being outcome-focussed and ultimately involves co-location of all of the key agencies involved in tackling DA (such as the police, child and adult safeguarding, health, specialist DA services and probation). The aim of this co-location approach is to improve the speed and effectiveness of information sharing. Each member of the family (perpetrator, victim and/or children) have a single point of contact-a ‘case coordinator’—who coordinates the case from start to finish and who seeks to maximise the possibility of effective interventions. The case coordinator is the same person for all family members and they work closely with relevant service providers (such as Probation and Children’s Services) to enhance the safeguarding provision for the whole family. The focus on victims, perpetrators *and* children ensures a holistic family approach. The development of this new approach was to address criticisms of the traditional MARAC process, overcoming issues with silo working, inconsistencies in the services that attended MARAC meetings and issues with practitioners adopting trauma-informed approaches to working with service-users (Walklate et al. 2021). In summary, the new system was designed on the following principles:

- All members of the process are central: the whole family approach.
- Only cases that meet the stated purpose of keeping people safe are prioritised.
- Speed–working closely with Children’s Social Care services, the model facilitates timely interventions by accurately assessing need and directing children, families and adults to appropriate support.
- Quick and effective co-located information sharing is prioritised, including case coordinator as a single point of contact to co-ordinate the process and ensure goals are met. Other staff are enabled and supported to make decisions and act quickly.
- The system is outcomes focussed–with determined outcomes being: reduced repeat offending; reduced domestic abuse referrals overall; speed of completion.

## Methodology

Our evaluation of the new MARAC process was conducted with the support of a policing partner in England. The evaluation incorporated three distinct administrative areas. The original methodological approach that had been planned involved a mixed methods approach, capturing a mix of original qualitative and quantitative data as well as a social return on investment (SROI) assessment. As we discuss throughout this article, there were delays in the launch and operationalisation of the new process, and this necessitated adapting our original research design to a process evaluation of the move towards the new process. The evaluation involved qualitative interviews with key stakeholders about the implementation process, online surveys distributed to core MARAC team members, perpetrators and victims-survivors, and observations of MARAC steering and working group meetings over a two-year period. Ethical approval was granted by [ANON] Ethics Committee prior to data collection. Steps to ethical adherence included informing participants about the purpose of the evaluation, how their anonymised responses would be used, and their rights to withdraw their responses for a limited period without implication.

Semi-structured interviews took place with fifteen stakeholders who were involved in the development of the principles underlying the new MARAC process. Although specific roles cannot be outlined here to ensure anonymity of participants, those interviewed spanned a range of areas including policing (varying role and rank) (*n* = 7), third sector (including DA services) (*n* = 4), health (*n* = 2) and probation (*n* = 2). For anonymity, participants have been given a unique identifier when quotes are used below. The main focus of the discussions during interviews were about the aims and perceived potential benefits of the new approach and process, as well as challenges of implementation. In addition, thoughts and perceptions about measuring and determining effectiveness and success were discussed. Recruitment for these interviews involved an email sent to all stakeholders involved in the development of the MARAC principles, followed by the option of an informal discussion with the researchers for those who sought further information as they considered their involvement in our evaluation. Practitioners who wanted to take part contacted the researchers directly via email.

Once the new MARAC process had been implemented in one clearly defined administrative area within the police force boundary for a period of six weeks, we were able to commence real time evaluation. The aim of the evaluation was to gather perspectives on the new system, harness continual learning, and progress improvement. During the evaluation period, 97 cases were administered.

In addition to the ongoing attendance at working group and steering group meetings and ongoing interviews, we administered a detailed anonymous survey (via MS Forms) to all of those who were involved with the implementation and roll-out of the new process. The survey included questions about how well the implementation had gone in the early weeks, perceived benefits, barriers and recommendations for further improvement of the new process. Respondents to this survey (*n* = 8) included representatives from police (*n* = 2), health (*n* = 1), adult and child safeguarding (*n* = 3), and probation (*n* = 2). We also circulated a second survey to all adult victim-survivors, and a third to perpetrators, thus all stakeholders experiencing the new process. Interestingly–and unfortunately–we received no responses to the latter two surveys in the short period of time we had left to complete our evaluation and we reflect on this in our discussion. This paper therefore reflects on the perspectives of practitioners who completed our online survey and those who took part in interviews.

Finally, over the two-year duration of the evaluation, evaluation team members, with the consent of all individual stakeholders, observed approximately twenty MARAC stakeholder and working group meetings. These meetings included discussions regarding planning and implementation of the new approach, logistics and any resistance to implementation. The meetings all took place via MS Teams and evaluation team members took ethnographic notes throughout their duration (totalling 30 h of observation). These are reflected on as appropriate throughout subsequent sections to add context where necessary.

The interview, survey and observation data were coded and analysed using thematic analysis (Braun and Clarke 2006) to identify overarching themes in the data. To enhance inter-rater reliability, two researchers performed this analytic stage where themes were independently identified within the data and then compared and discussed to reach a thematic consensus. Themes were then applied throughout the data including the interviews, survey and observation analysis. In what follows, we discuss our findings according to two principal themes namely, ‘benefits of adopting the new process’ and ‘challenges of implementing the new process’.

### Findings

The results we present report practitioner perspectives on the new outcome-focussed process aimed at providing holistic support for victims, children and perpetrators—‘whole family’ approach. Despite a shared vision and enthusiasm to adopt the new process across all agencies, there were challenges and barriers to its successful implementation.

#### Benefits of the new process

Interviewees discussed the improvements to, and benefits of, the new multi-agency way of working in detail and depth. Two of the most common responses were to detail the advantages of more proximate and close partnership working (including core partner representation and speed of processing and actions) and lack of duplication of work (multiple benefits for several stakeholders).

One respondent to the online survey best illustrates the streamlining of the new process and what works well:

Referrals are heard on a weekly basis and we hold two meetings per week. Good representation from Core Partners representing several agencies. Partnership approach to reducing risk with clear actions, roles and responsibilities identified and engagement of the whole family. We review each open case on a fortnightly basis and avoid cases drifting within the system. Effective, joined up safeguarding approaches are in place. (Survey respondent, police).

Relatedly, all interviewees reflected on the ways in which the centralisation of multi-agency working in the new approach avoided duplication of efforts by professional and victim-survivor stakeholders. Much of these positive reflections centred on the new ‘case coordinator’ role, whereby each family has a dedicated person who oversees the communication and support provision for all parties. This is reflected in the following quote:

It also avoids duplication, because if we have this coordinator that means the victim doesn’t need to tell her story 7 times for instance. The key thing is not to scratch the surface, but look at the root cause for everyone, and making sure everyone is safeguarded. (Interview 7, health)

Participants felt that having this coordinated approach therefore enhanced whole family engagement with the support provision. Furthermore, several respondents elaborated in the survey and interview discussions on the new system being more focussed on team working, reducing ‘silo’ working, as well as the timeliness of communications and speed. For example one survey response articulated this as follows:

Victims’ voices are being heard much quicker. It has reduced the feeling of working in silo which can be the case sometimes. Partner agencies are more mindful of other agencies’ timescales. (Survey, third sector)

Another interviewee explained these benefits to us in the following way:

So we could go to the victim directly straight away and understand what they needed, working with the family holistically, whether they want to stay together or not. It helped to focus on the views of the victim and can bring in all those agencies to support the family together. It’s much better than working in silos. (Interview 5, police)

Other participants described how the focus on outcomes worked to the benefit of all:

The new model showed me was that it was people-focussed, almost like we were beginning at the end. We were looking at what the outcome should be first, and then deciding what the whole family wants, to get the right things in place for them. (Interview 6, third sector)

The new multi-agency response is really good because, they sometimes wait for multi agencies for hours and hours before they can do anything. I remember when I was on the custody process team, we were trying to get a refuge for a lady who didn’t have recourse to public funds. Normally you have to go to social care first and they would pay for the refuge but because social care are now on the review team they got it with the click of a finger (Interview 1, police)

This collectively highlights that the new process not only improved the timeliness and resource management from the perspective of the MARAC team, but it also improved service delivery and the support provision provided to the whole family. Furthermore, five participants reflected on these benefits from the perspective of improving the financial cost implications of the MARAC process. One quotation from an interviewee described this in terms of longer-term value for money:

I think it’s expensive work but the expense will completely outdo the drain. If you consider the admin costs are estimated at a million pounds for the year, but if you look at the statistics of the cost of domestic abuse and mental health, everything around it and young people it is such a shame that the finance isn’t given to that. (Interview 12, police)

The way the interviewees alluded to the financial benefit captures a hard to measure feature of any multi-agency provision including innovative multi-agency partnerships to tackle DA. Social returns on investments in these contexts are notoriously difficult to conduct in a meaningful manner, especially when conducting snapshots of short-term or early stages of innovative practice. In addition, softer qualitative measures of victim satisfaction and longer-term perpetrator behaviour change are hard to quantify. The complex dynamics that give rise to DA cannot be isolated in traditional ways of counting and measuring crime reduction outcomes. We return to this in our discussion.

All participants mentioned the benefits for individual family members involved in the process: victim/s, perpetrator/s and child/children. One respondent’s observation was atypical and signifies their amazement that, on overhearing a telephone conversation (due to the close co-location environment), perpetrators’ needs were being addressed:

I did observe a telephone call with a perpetrator and that amazed me that they were asking the perpetrator what they wanted to change and it turned out that the perpetrator had been sent to services that weren’t helping him but he wanted a different service. At the time it seemed like a lightbulb moment because previously it had been repeat after repeat but they hadn’t actually asked the person what they want. (Interview 10, health)

Findings about the perpetrator as stakeholder were generally less enthusiastic and we report on this in the next section and also return to this in our discussion. Furthermore, as well as the benefits of the process itself, participants raised various challenges with implementation.

#### Challenges of implementing the new process

We can collapse the various challenges relevant to the complex work of implementing a new approach to multi-agency policing of DA under three sets of issues. First, there were anxieties and concerns about the assessment of risk. Second, there were common concerns noted about the perpetrator as a key stakeholder in the process and third, there are challenges that focus on the experience of the planning, launch, and operationalisation of the new process. This third area is where, as process evaluators and ethnographic observers, we can provide additional commentary, to feed in to our broader discussion. We discuss each of these challenges in turn.

*Risk*: Firstly, accurately capturing and focussing on risk whilst engaging in a trauma-informed, holistic way of supporting families was described as a particular challenge when implementing the new MARAC process. For example:

I think there is an ongoing piece of work to do around how do you take a trauma involved approach to this because naturally you slip into covering your back, checking that you have done everything and doing your basic safeguarding thing and that’s the easy thing to do [. . .] Because this isn’t about being kind to people for kindness sake, you need to be kind to people at the moment you are engaging with them, as it is kindness and compassion that moves people forward. But doing that within a risk framework. It’s not easy. (Interview 11, third sector)

The issue with just looking at risk and focussing on high-risk cases, is you inevitably focus on what is going on at that time without thinking about the broader context of what that woman has going on in her life. Risk changes all the time, you could have a case that comes in where a police officer grades it as standard, but it isn’t standard because when you look at the history of the perpetrator, you know they are not standard risk. But you almost feel bound by this idea of risk (interview 9, third sector)

One survey responder suggested:

I think there is a need for training on risk and what meets the criteria, we are being told that it is just for the most high-risk cases, which has always been the criteria. It is how we identify these risks within the new MARAC and still capture the quick relationship, coercive control, ongoing abuse, repeat cases which are shown time again to occur (not high levels of physical abuse). This is under discussion and training is being looked at. The concept is great but risk cannot always be managed by a focus on support and health, especially in the cases of non-compliant repeat offenders. (Survey, police)

There are at least two key issues at play here. First, the disjuncture between assessing risk, as required within any MARAC process, but doing so whilst using a holistic, trauma-informed approach. Risk is a structurally neutral concept which does not account for the ways in which intersectional constraints (such as gender and ethnicity) can impact behaviour (Mythen 2014), therefore adopting a risk-focussed approach whilst attempting to be trauma-informed creates a fundamental tension (see inter alia Hannah-Moffatt 2015). Secondly, understanding which cases are considered as part of the MARAC process, with a recognition that many cases of DA may not be assessed as ‘high risk’ using tools such as the DASH, yet may feature highly risky behaviours constituting coercive control (Barlow and Walklate 2021; Walklate et al. 2021).

*Working with perpetrators*: Similarly, there were many comments about the difficulty of providing holistic support for perpetrators, both in reaching them and finding accessible services to address their needs:

There’s always a level of support for victims and children but support for perpetrators is poor. Social workers are not confident or well equipped. There are few courses for perpetrators to go on and a low take up. (Interview 8, probation)

So it was a challenge to look at things from a perpetrator perspective. For years I had been saying if we don’t work with perpetrators, that perpetrator, without intervention will move on to someone else. But speaking to perpetrators was a challenge. It was hard to speak to them with a different hat on if that makes sense? But it is necessary. (Interview 9, third sector)

These observations have significant implications for the adoption of a genuine whole family approach, as working with perpetrators has increasingly been recognised as an important component in tackling and preventing DA (Renehan 2021).

*Practical complexities:* The third challenge that was raised by participants concerned implementation issues. One example included the sheer number of partner agencies involved in the process:

We underestimated the complexity of involving multiple partners and fundamentally changing how they do a key activity. We are trying to get to a position where they can agree a model to improve the services. (Interview 3, police)

Once it was established that we needed to set up a flagship model and start taking a small number of cases, get the multi-agency partners in, let them see the work we are doing - then we would be able to work out slowly how many staff we would need to implement the model. But then COVID-19 came along and that was put to the side. (Interview 7, health)

There are two issues at play here. The first relates to partner agencies understanding and appreciating the roles and challenges of the various agencies involved in the process. The second was the issue of the COVID-19 pandemic significantly impeding the delivery of the new process. Although the pandemic enhanced the online delivery of MARAC’s nationally (Walklate et al. 2021), this move to online working stands in stark contrast to the proposed co-location of agencies in one building in each geographic area as proposed in the new process. Furthermore, attempting to set up a new approach during the unprecedented global pandemic, particularly when it was as complex as innovating multi-agency responses to policing DA, was unsurprisingly difficult. Collectively, these kinds of challenges led to significant delays in the implementation process, with only one district within the evaluation area implementing the new process during the evaluation period as previously noted.

Furthermore, there were also practical issues identified in finding an appropriate shared IT system that could be used by all agencies. For instance, “we had to revolutionise our whole IT system. When the team first came over they were using paper” (Interview 1, police). Another participant stated:

I’m still not sure we have found a proper system that can include everyone. It is often health that is the issue on this. For a long time we were using this huge excel spreadsheet which just isn’t sustainable (Interview 5, police)

A lack of a shared IT system is often a barrier for successful information sharing between agencies (Sloper 2004). Improved information sharing was a key motivator for developing the new MARAC process, therefore the urgency in finding an appropriate solution to this was recognised by participants.

In sum, the reflections of participants highlight that despite all participants emphasising the value of the new process in improving the service delivery of the MARAC for professionals and the whole family, implementing such innovations in practice is a significant challenge and requires investment in the form of financing, time, and belief in the new process itself.

## Discussion

Our discussion considers three sets of implications arising out of our findings. First, we extend our discussion of the implications for innovative multi-agency partnership policing of DA. Second, we make some specific observations about the perpetrator as stakeholder. Third, our discussion considers some implications for research including co-production, professional experience as evidence and innovative methodologies.

### Implications for innovative multi-agency partnership policing of domestic abuse.

The findings shared here highlight that despite all stakeholders reflecting on the clear benefits of adopting a holistic, whole family approach to the MARAC process, there were both practical and conceptual challenges in implementing this type of innovative multi-agency response. On a practical level, the implementation of the new process took place at a time during both a global pandemic and also an ongoing period of austerity, when there had been significant funding cuts to public services and resources. Therefore the delays in implementing the new process and issues with identifying appropriate shared IT systems need to be situated within this context. The issues with this are evidenced by only one of the three districts being in a position to implement the new process within the timeframe of the evaluation.

There were various facilitating factors for the area that was able to implement the new process during the evaluation period. These included having at least one key person driving and coordinating the implementation of the new process, and adopting a flexible approach to delivery where possible. On the latter point, during the meetings that the project team observed, it was consistently recognised that each administrative district would need to adapt the delivery of the new process due to the nuances and specificities of relevance to each geographic area. Adopting a ‘one size fits all’ approach is not the best way of innovating within the context of DA (Walklate et al. 2021). This point is clearly illustrated here, as there were three implementation models that were required for each administrative area, due to differences in population size, diversity, and specificities of service provision. However, there was also recognition in the meetings we observed that any changes to delivery should not detract from the overarching aims and core principles of the new process. A tricky balance to strike when this is situated within the broader context of austerity and increased pressure to respond more effectively to DA, particularly for the police (HMIC 2015).

Furthermore, despite all interviewees reflecting positively on the benefits of the new process, our observations of meetings demonstrated some issues with inertia for those who had been working in the field for some time (See also Balderston et al. 2019). There was at times some resistance or fear in adopting new ways of working expressed by these individuals. In particular, there was a tendency for these individuals to focus inwardly at the needs and pressures of their own agency/ organisation, rather than understanding the complexities across the multiple agencies involved in the process. For example, one of the administrative districts that had difficulties in navigating the implementation process faced particular issues with colleagues who worked in health and the police often having competing interests and priorities (see also Humphreys et al. 2018). This could be related to resistance to cultural change due to established ways of working and resistance to externally imposed change (Houtsonen et al. 2020).

As well as facing practical issues, there were also difficulties in implementation caused by conceptual tensions, particularly in relation to risk. The difficulties in translating the concept of risk to responses to DA, particularly process-driven offences such as coercive control, have been discussed at length elsewhere (Barlow and Walklate 2021). However, of particular pertinence to this paper are the inherent tensions in adopting a risk framework when aiming to provide trauma-informed, whole family responses to DA. DA risk assessment tools usually focus on responding to ‘incidents’ and identifying risks in the ‘here and now’, rather than considering any longer-term safety implications (Barlow and Walklate 2021). Holistic, trauma-informed responses are focussed on looking beyond the ‘present’ context and focus on developing individualised approaches to dealing with traumatic experience. There is therefore a clear incongruence between adopting both a risk and holistic, trauma-informed response to DA simultaneously. These conceptual nuances are important, because as reflected on by participants, these tensions can lead to a lack of shared understandings in definitions, approach and priorities, which are key components of any successful policing multi-agency response to DA (Cleaver et al. 2019).

In our experience of evaluating a range of multi-agency processes and innovations all professional practitioner stakeholders tend to appreciate their potential capacity to prevent and protect in their endeavour to tackle DA such that women and children are safer, and men are held to account by pre-emptively addressing their complex needs. However, safeguarding from abuse presents tensions around risk. The prevent strand of such a strategy sits, and is operationalised, alongside the support and service provision or protect strand. The former and the latter are designed to do two things simultaneously—tackle perpetrators and support victims. This ‘holistic’ strategy can present challenges for DA partnership work (Davies and Biddle 2017a, b; Davies 2018). Concerns about victims’ safety and risk are well founded. The imbalance of power in abusive relationships is the crux of the problem.

Furthermore, all agencies involved in multi-agency DA responses may use different risk assessment tools, or at the very least assess and understand risk in different ways, meaning it is unlikely that all agencies involved in the new MARAC process will have a shared understanding of risk. These issues are also evident in the work of Barlow et al. (2021b), highlighting that within the context of policing alone, there are at least four different ways of assessing risk in DA cases, comprising of at least two risk assessment tools (i.e. call handler and front-line level). Continuing to place the concept of risk as a central component of the new process may therefore inhibit the potential to adopt a genuine holistic and multi-agency approach to responding to DA. One possible solution could to be de-centralise the concept of risk and focus on safety which would allow the voices and preferences of victim-survivors to be heard more explicitly. There is arguably no better assessor of their own safety than victim-survivors themselves (Barlow and Walklate 2021), so a de-emphasis of the concept of risk may pose one possible solution to address these conceptual issues.

All stakeholders in innovative multi-agency partnership processes and approaches to be convinced and confident there are no escalated risks to victims’ safety. The critical mass of stakeholder views are healthy reminders of how highly volatile, threatening, and risky domestic situations can be and how women, who are separated from their violent partners, are at risk of post separation fatal violence (Davies 2018). Safeguarding from abuse presents tensions between risk assessment and a trauma-informed whole family approach and such tensions are rightly at the heart of the hard and complex work of implementing new multi-agency risk assessment approaches to policing domestic abuse.

### Perpetrators as stakeholders

Despite aiming to be a ‘whole family’ approach, participants reflected that perpetrators were largely absent from the support provision provided in the new MARAC process. There were some positive reflections shared, as noted earlier in the paper, but generally it was felt that support for perpetrators was lacking. This ranged from participants acknowledging that work with perpetrators ‘required more thought and attention’ (interview 3, police), to a more explicit reflection of ‘we are currently failing perpetrators in the support provision we offer’ (interview 8, probation). There is increasing recognition that DA cannot be reduced or prevented if working with perpetrators is not appropriately invested in and resourced, but the complexities of doing so within a multi-agency context are extensive (Renehan 2021).

Criticism of how perpetrators of DA are managed by CJ agencies have persisted for decades (Buzawa and Buzawa 2003; Burman and Brook-Hay, 2018). Although the provision of perpetrator programmes has grown in a UK context and other areas across the global north, their efficacy is still subject to considerable debate, particularly programmes which do not utilise a strengths-based and feminist orientated approach (Renehan 2021; Downes et al. 2019). The Domestic Abuse Act (2021) explicitly acknowledges the need to engage more critically with perpetrators of DA. However, it is crucial that such interventions are adequately resourced, responsive to a diverse perpetrator population, and where possible, delivered as part of a holistic, trauma-informed, multi-agency package (Kelly and Westmarland 2015; Renehan 2021).

Furthermore, it is pertinent to note that as part of our evaluation, we sought the perspectives of victim-survivors and perpetrators regarding their views on the new MARAC process. However, we received no responses to our short survey. All case coordinators circulated the survey to victim-survivors and perpetrators. There are many and varied potential reasons for this lack of response, such as a lack of time or capacity to complete given the likely distressing nature of their personal situation and as noted, we had to start this phase of the evaluation much later than planned due to the implementation barriers, therefore the window for completing the survey was shorter than we hoped. Understanding levels of satisfaction or otherwise for victim-survivors and perpetrators is hugely significant when attempting to measure success in evaluations of new processes, yet identifying the best way to do this is complex. DA is highly complicated and success cannot be measured in terms of measuring crime reduction outcomes or as one participant notably said ‘success isn’t a reduction in cases coming through the MARAC, I would be worried if that happened’ (interview 6, third sector). One way of maximising the possibility of success for the new process is ensuring that all stakeholders are engaged with and supported, therefore working with perpetrators using trauma-informed approaches is an important step for potential success for the whole family approach.

### Implications for research

As noted earlier, how to evaluate and how to measure effectiveness remains a knotty problem. As was the case in the early days of multi-agency and partnership working, there remains considerable incentive to demonstrate success, and balanced evaluation of crime prevention initiatives continues to be difficult. In particular, the difficulty of teasing out the influences of alternative strands to any one initiative is especially problematic. Tenders for evaluations are often extremely demanding in terms of their requirements to assess outcomes. Given the numerous aims of multi-agency work which, in the context of policing DA, will often have tri-partite ambitions to prevent and reduce offending and victimisation, tackle perpetrators and support victims all under one shared vision evaluation and SROI assessments are fraught with difficulty. Within these various strands of the muti-agency work what constitutes success/failure is often poorly defined. Hard rather than soft measure outcomes are often taken as more robust evidence, yet what they are evidence of is often unclear. Softer satisfaction and qualitative measures often tend to further complicate the overall picture thus what works element remains dubious. The incentive to be successful remains a key driver (Davies 2022).

A Social Return on Investment (SROI) is a framework for measuring and computing a composite value of an investment. In the context of the social sciences it is a study where the method of analysis includes both qualitative and quantitative data and follows a set of standardised principles to enable the social, personal, and environmental outcomes of a service or new process to have a monetary value assigned to them. Such a study aims to capture the story of change by measuring social, environmental, and economic outcomes and using monetary values to represent them. A ratio of benefits to costs is then calculated such that the social value worth is represented in £. In the context of policing domestic abuse the aim it to demonstrate the positive or negative financial impacts for victims, perpetrators, and a range of stakeholders.

Whilst SROI studies have been commissioned in connection with developments in the criminal justice context and a few in the context of poling domestic abuse, the reports remain separate to other evaluations and research. One article from 2013 (Jardine and Whyte 2013) reports that whilst SROI analysis can prove a valuable exercise charities and third sector organisations are unlikely to be able to collect data sufficient to establish causal links between their interventions and the complex processes and innovations they seek to measure. The authors also report that third sector organisations are concerned about how the results of any social impact measurement would be used by others. Such organisations reportedly admit that negative results remain unpublished propelling organisations to produce similarly positive results with less emphasis on the limitations. Some literature describes efforts to tackle specific concerns, such as provision of support for perpetrators (Ariss et al. 2017), improving access to non-police services (Koppensteiner et al. 2019; Balderston et al. 2019), improving inter-agency communications systems (Vogt 2021), and releasing time and resources including dedicated staff members to MARAC processes (Hamilton et al. 2021). All of these sources recommend continuous development and evaluation of evolving models.

## Conclusion

In the period since multi-agency working became the dominant approach to tackling DA, there has been continuous innovative practice impacted in part by legislative developments. However, little is known about what tends to enhance or inhibit the roll-out of such initiatives and incremental developments. We have focussed on the process element of multi-agency working to tackle DA. In imagining a flagship multi-agency model for policing DA, we suggest there is a need to more thoroughly consider the process and implementation problem. Drawing on recent and previous process evaluation experiences of innovative multi-agency ways of working, we have reported the positive benefits of evolving processes from a practitioner perspective. These include a more streamlined MARAC system that demonstrates the benefits of co-location, co-produced innovation, and taking a whole family approach. We recommend further research that gleans the perspectives of families and perpetrators to provide insight and balance to the evidence base. We have also outlined the challenges in respect of perpetrators as stakeholders and the problem of assessing the dynamics of risk. The latter part of our discussion calls for enhanced planning and co-production in research on ways to effectively enhance the policing of DA. This may entail greater consideration of professional experience as valuable evidence and co-devising of realistic evaluation scripts between commissioners and researchers.
